# Impact of the COVID-19 pandemic on symptoms of anxiety and depression and health-related quality of life in older patients with chronic kidney disease

**DOI:** 10.1186/s12877-021-02593-0

**Published:** 2021-11-19

**Authors:** C. G. N. Voorend, M. van Oevelen, M. Nieberg, Y. Meuleman, C. F. M. Franssen, H. Joosten, N. C. Berkhout-Byrne, A. C. Abrahams, S. P. Mooijaart, W. J. W. Bos, M. van Buren, on behalf of the POLDER investigators, Arjan van Alphen, Noeleen Berkhout-Byrne, G. F. van Breda, Marjolijn van Buren, Henk Boom, Willem Jan Bos, Adry Diepenbroek, Marielle Emmelot-Vonk, Casper Franssen, Carlo A. J. M. Gaillard, Nel Groeneweg-Peeters, Bettie Hoekstra, Nienke Hommes, Francoise Hoornaar, Hanneke Joosten, Joep Lagro, Elisabeth Litjens, Femke Molenaar, Simon P. Mooijaart, Aegida Neradova, Mike Peters, Wilma Veldman, Carlijn Voorend, Lidwien Westerbos, Carlijne Westerman-van der Wijden, Judith Wierdsma

**Affiliations:** 1 Department of Internal Medicine, Leiden University Medical Center, Leiden, The Netherlands; 2 Department of Clinical Epidemiology, Leiden University Medical Center, Leiden, The Netherlands; 3 Department of Nephrology, University Medical Center Groningen, University of Groningen, Groningen, The Netherlands; 4 Department of Internal Medicine, Division of General Internal Medicine, Section Geriatric Medicine, Maastricht University Medical Center+, Maastricht, The Netherlands; 5 Department of Nephrology and Hypertension, University Medical Center Utrecht, Utrecht, The Netherlands; 6 Department of Gerontology and Geriatrics, Leiden University Medical Center, Leiden, The Netherlands; 7 Department of Internal Medicine, St. Antonius Hospital, Nieuwegein, The Netherlands; 8 Department of Internal Medicine, Haga Teaching Hospital, The Hague, The Netherlands

**Keywords:** Aged, Chronic kidney diseases, Coronavirus disease-2019 (COVID-19) pandemic, Mental health, Quality of life

## Abstract

**Background:**

Older patients with advanced chronic kidney disease are at increased risk for a severe course of the coronavirus disease-2019 (COVID-19) and vulnerable to mental health problems. We aimed to investigate prevalence and associated patient (demographic and clinical) characteristics of mental wellbeing (health-related quality of life [HRQoL] and symptoms of depression and anxiety) before and during the COVID-19 pandemic in older patients with advanced chronic kidney disease.

**Methods:**

An ongoing Dutch multicentre prospective cohort study enrols patients of ≥70 years with an eGFR < 20 mL/min/1.73m^2^ from October 2018 onward. With additional questionnaires during the pandemic (May–June 2020), disease-related concerns about COVID-19 and general anxiety symptoms were assessed cross-sectionally, and depressive symptoms, HRQoL, and emotional symptoms longitudinally.

**Results:**

The 82 included patients had a median age of 77.5 years (interquartile range 73.9–82.1), 77% were male and none had tested positive for COVID-19. Cross-sectionally, 67% of the patients reported to be more anxious about COVID-19 because of their kidney disease, and 43% of the patients stated that their quality of life was reduced due to the COVID-19 pandemic. Compared to pre-COVID-19, the presence of depressive symptoms had increased (11 to 22%; *p* = .022) and physical HRQoL declined (*M* = 40.4, *SD* = 10.1 to *M* = 36.1, *SD* = 10.4; *p* < .001), particularly in males. Mental HRQoL (*M* = 50.3, *SD* = 9.6 to *M* = 50.4, *SD* = 9.9; *p* = .913) and emotional symptoms remained similar.

**Conclusions:**

Older patients with advanced chronic kidney disease suffered from disease-related anxiety about COVID-19, increased depressive symptoms and reduced physical HRQoL during the COVID-19 pandemic. The impact of the pandemic on this vulnerable patient group extends beyond increased mortality risk, and awareness of mental wellbeing is important.

**Trial registration:**

The study is registered at the Netherlands Trial Register (NTR), trial number NL7104. Date of registration: 06-06-2018.

**Supplementary Information:**

The online version contains supplementary material available at 10.1186/s12877-021-02593-0.

## Introduction

Older persons are highly vulnerable during the coronavirus 2019 (COVID-19) pandemic: previous research showed a twenty-fold increased mortality in people aged 80 years or above compared to 50–59 year olds [1]. Besides, the emergence of the COVID-19 pandemic resulted in increased symptoms of both anxiety and depression in the general population, outpatients, and healthcare workers [2–4]. Also, mental distress during the COVID-19 outbreak was higher than before the pandemic [5] and seems to persist as the pandemic continues [6]. There are concerns that the social restrictions for COVID-19 disproportionately affect older people, mainly by increased (feelings of) social isolation and loneliness [7, 8], which, in turn, may increase patients’ risk of anxiety and depressive disorders [9].

Patients with advanced chronic kidney disease (CKD) have shown to be more vulnerable to mental health problems, such as anxiety and depression [10]. In this population, emotional symptoms, like symptoms of anxiety and depression, worrying, sleep disorders, perceived lack of social support, and reduced social interactions, affect not only health-related quality of life (HRQoL) [11–14], but have also been associated with adverse clinical outcomes and increased mortality risk [15, 16]. Above and beyond, the presence of CKD is an important risk factor for a more severe course of COVID-19, as patients with advanced CKD (stage G4/G5; severely decreased glomerular filtration rate or kidney failure) have a 2.5 times increased mortality risk, compared to those with normal kidney function [1].

To date, no studies have been performed on mental wellbeing in older patients with advanced CKD during the COVID-19 pandemic. Therefore, our goal was to explore mental wellbeing in older patients (aged ≥70 years) with advanced CKD (estimated glomerular filtration rate [eGFR] < 20 mL/min/1.73m^2^). First, to investigate kidney disease-related concerns about COVID-19 and general anxiety symptoms. Second, to explore changes in the presence of depressive symptoms, HRQoL, and emotional symptoms during the COVID-pandemic. Third, to examine if these (changes in) mental wellbeing are associated with patient (demographic and clinical) characteristics.

## Materials and methods

### Study population

The present study uses patients from the *Pathway for OLder patients reaching End-stage Renal disease* (POLDER) study cohort. POLDER is a currently ongoing multicentre prospective cohort study (Netherlands Trial Register identifier NL7104) which investigates the feasibility of implementing a standardised geriatric assessment into Dutch routine care for older patients with CKD stage G4/G5. Participants in POLDER are enrolled from October 2018 onwards in four non-academic and five academic Dutch nephrology units. The inclusion criteria are patients aged 70 years or older with an eGFR below 20 mL/min/1.73m^2^ and the ability to read and understand the questionnaire. In POLDER, geriatric assessment is conducted at inclusion and after a one-year follow-up.

For the current study, all patients participating in the POLDER study were eligible if not lost to follow-up or deceased. An additional inclusion criterion was the completion of a baseline assessment before March 1st, 2020. This date was chosen to ensure that at least one assessment was completed before the World Health Organisation declared the COVID-19 outbreak a pandemic (11th of March) and the Netherlands went into lockdown (15th of March), which involved the closing of restaurants, educational institutions, sporting and cultural facilities, working from home and obliged social distancing. Patients were excluded if they received a kidney transplant after enrolment into the POLDER study.

The research was conducted according to the principles of the Declaration of Helsinki and the Dutch Medical Research Involving Human Subjects Act (WMO), and approved by the Medical Research Ethics Committee Leiden-Den Haag-Delft (reference NL65322.098.18). All participants provided written informed consent when entering the POLDER study.

### Study design

This current multicentre prospective cohort study consists of two parts: 1) a cross-sectional part using data derived from questionnaires that were not yet included in the POLDER study (i.e. which were specifically sent out and collected for this current study), and 2) a longitudinal part using data from questionnaires that were already included in the POLDER study.

### Data collection

In the POLDER study, baseline data was collected between November 2018 and March 2020 using self-reported questionnaires, interview-based questionnaires collected in the hospital, and data from electronic patient files. Demographic and clinical characteristics collected at baseline included age, sex, living status (living together versus alone), level of education (high versus low), eGFR, Charlson comorbidity index [17], and Clinical frailty scale [18]. For the current study, additional questionnaires were sent by mail between May 19th and June 23th 2020 (during the COVID-19 pandemic) together with an information letter about the additional study procedures. All questionnaires included are described below. If no response was received after two weeks, patients were contacted by phone and asked if they were willing to fill in the supplementary questionnaire. Also, patients’ most recent eGFR, provisional dialysis initiation date and modality (haemodialysis / peritoneal dialysis) was collected from their treating physicians. All data was collected in a database managed by Nefrovisie, the Dutch quality institute for nephrology.

### Outcomes measured during the COVID pandemic (cross-sectional)

#### Kidney disease-related concerns for COVID-19

Participants were asked to respond to four statements that were created specifically for this study: (1) ‘*I am more anxious about the coronavirus because of my kidney disease’,* (2) ‘*I experience more stress from the coronavirus because of my kidney disease’*, (3) ‘*I feel more down because of the coronavirus’*, and (4) ‘*I experience a lower quality of life due to the coronavirus’*. Patients’ level of agreement with the statements was rated on a five-point Likert scale, ranging from 1 ‘totally disagree’ to 5 ‘totally agree’. Patients were considered to have concerns if they scored 4 ‘agree’ or 5 ‘totally agree’.

#### General anxiety symptoms

Anxiety symptoms were assessed using the anxiety subscale of the Hospital Anxiety and Depression Scale (HADS-A) [19]. Seven items are scored from 0 (not present) to 3 (considerably bothersome), assessing predominantly psychological rather than somatic anxiety symptoms, for example ‘*I have lost interest in my appearance*’. The item scores are summed to provide the HADS-A sub-scores, with scores ranging from 0 to 21. HADS has proven reliability and validity across different ages [20] and is validated in dialysis patients [21]. A score of ≥8 is an often-used cut-off score as indication of anxiety [22].

### Outcome measures measured prior and during the COVID-19 pandemic (longitudinal)

#### Depressive symptoms

Depressive symptoms were assessed in two steps. First, patients were screened with two Whooley-questions (i.e. ‘*During the past month, have you been bothered by feeling down, depressed or hopeless?*’ and ‘*During the past month, have you often been bothered by little interest or pleasure in doing things?*’) to assess the presence of a depressed mood and anhedonia in the previous month [23]. Second, with one or two confirmative answers, the fifteen-item Geriatric Depression Scale (GDS-15) was completed as well [24]. Each binary item (e.g. ‘*Do you often feel helpless?*’) can be scored one point (0 ‘not present’, 1 ‘present’). The total score ranges from 0 to 15, and a cut-off score of ≥5 is often used to indicate depression [25]. Both questionnaires have been validated and widely used in older adults [25, 26] and in some CKD populations [10].

#### Health-related quality of life

The twelve-item Short Form Health Survey (SF-12) measures HRQoL. Using several questions on mental and physical health (e.g. ‘*Does your health now limit you in climbing several flights of stairs? If so, how much?*) and a scoring algorithm, the Mental Component Summary (MCS) and Physical Component Summary (PCS) scores are calculated. Scores for both Component Summaries are transformed to a scale ranging from 0 to 100, with a mean of 50 and a standard deviation (SD) of 10 in the general United States population, where higher scores reflect better HRQoL [27]. The SF-12 has proven applicability in patients with CKD and has adequate psychometric properties to display changes in HRQoL [28].

#### Emotional symptoms

Six symptoms were derived from the Dialysis Symptom Index (DSI); i.e. *feeling anxious, feeling sad, worrying, feeling nervous, trouble falling asleep, trouble staying asleep* [29]. Per present symptom, burden was scored on a five-point Likert scale ranging from 1 ‘not at all bothersome’ to 5 ‘very bothersome’.

### Statistical analysis

First, descriptive statistics were computed to describe the demographic and clinical patient characteristics, concerns about COVID-19, and general anxiety symptom score (HADS-A). Proportions were reported for nominal and categorical data, means (M) with standard deviations (*SD*) for continuous normally distributed data, and median with interquartile range (IQR) for skewed data.

Secondly, data from prior to the COVID-19 pandemic was gathered from the most recent assessment before the pandemic, i.e. either the initial or follow-up measurement of the POLDER study. To assess whether depressive symptoms (GDS-15 score and GDS-score ≥ 5), HRQoL (MCS, PCS scores), and presence of six emotional symptoms changed during COVID-19 pandemic, we conducted Wilcoxon signed-rank tests for non-normally distributed outcomes, McNemar tests for binary outcomes, and paired t-tests for normally distributed outcomes.

Third, we investigated the associations between demographic and clinical (age, sex, education, living status, frailty, comorbidity, eGFR, kidney replacement therapy, follow-up duration) patient characteristics, and disease-related COVID-19-anxiety, general anxiety symptoms, change in depressive symptoms (GDS-15 score) and change in HRQoL (MCS-12, PCS-12 scores). We tested correlation with Kendall’s tau-b for continue independent variables, Mann-Whitney-U tests for binary independent variables with non- normally distributed outcomes, and used unpaired t-tests for binary independent variables with normally distributed outcomes. Overall, a two-sided significance level (alpha) of .05 was used. All analyses were conducted with SPSS 25.0.

#### Missing values

Missing values of SF-12 questions were, if less than 50% missing, imputed using multiple imputation techniques (i.e. maximum 50 iterations, five imputations (repetitions) and predictive mean matching [30]). Other missing variables were not imputed, as these were reasonably complete.

## Results

**Fig. 1:**
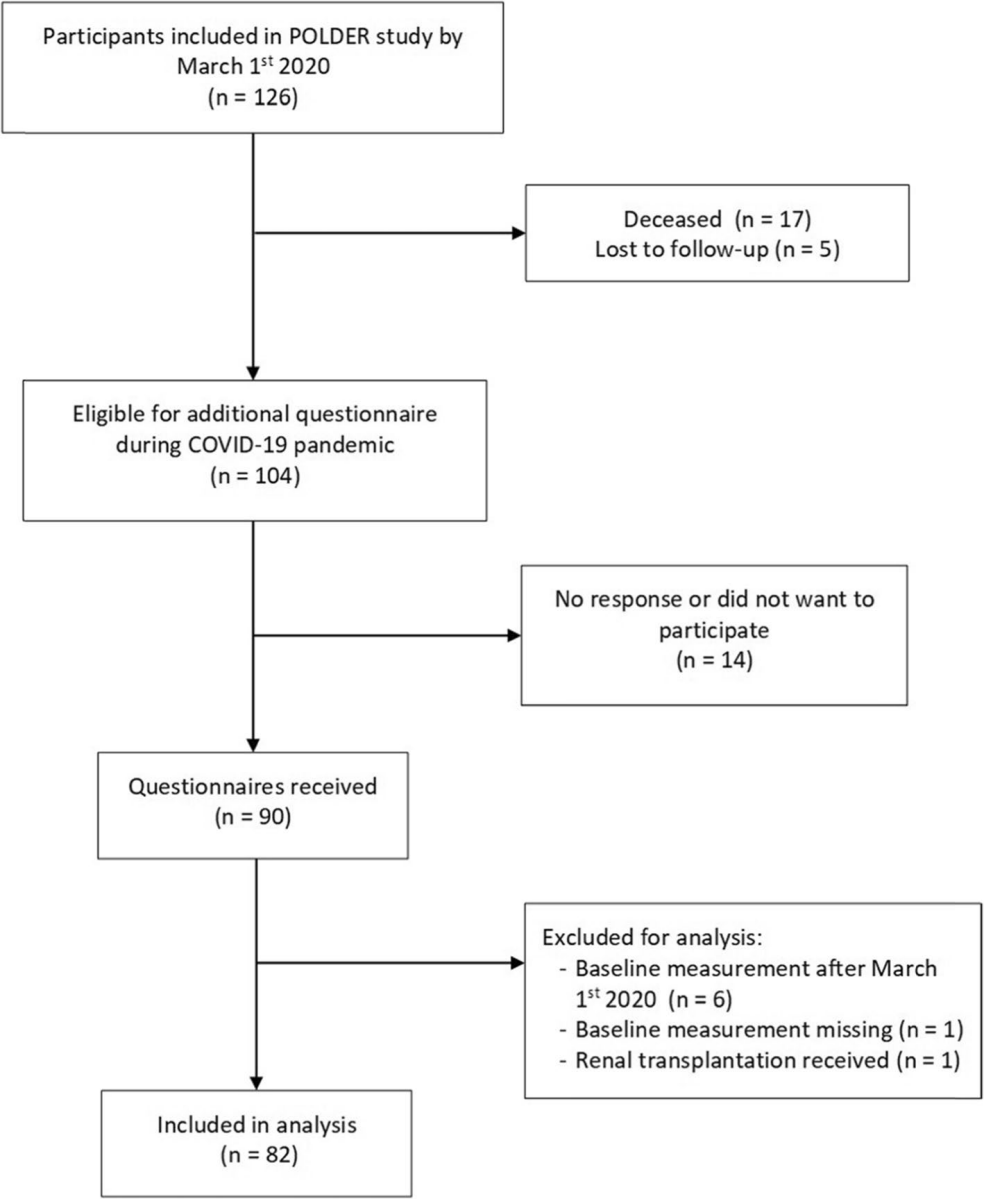
Sampling flow chart

Out of the 126 patients in the POLDER study, 104 eligible participants were sent the supplementary questionnaire during the COVID-19 pandemic (Fig. 1). Ninety eligible patients returned the questionnaire (response rate 87%). Non-responders did not differ in baseline characteristics from the study cohort (data not shown). Eight participants were excluded from analysis because no baseline assessment was available or they received a renal transplant. In total, 82 patients were included. Participant characteristics are shown in Table 1. The majority of participants were male (77%) and ranged in age from 70 to 95 years (median 77.5, IQR 73.9–82.1) at baseline. None of the participants had tested positive for COVID-19. In total, ten patients (12%) had started dialysis treatment; two (3%) before baseline of the current study and eight (10%) during follow up. For non-dialysis patients, eGFR declined with a mean of 1.1 (*SD* = 3.7, *p* = .012) mL/min/1.73 m^2^ during follow-up. The median time between assessments prior and during the COVID-19 pandemic was 8.0 months (IQR 4.7–13.2).

**Table 1:** Participant characteristics prior to the COVID-19 pandemic

Participant characteristics (***n*** = 82)	Prior COVID-19 pandemic
Age, median (IQR)	77.5 (73.9–82.1)
Sex, male (%)	63 (76.8)
Living/marital status^a^	
Living together with partner / married	52 (63.4)
Living alone / widow	29 (35.4)
*Missing*	*1 (1.2)*
Level of education (%)^a^	
Low (none, primary, secondary education)	51 (62.2)
High (vocational, higher secondary, tertiary)	29 (35.4)
*Missing*	*2 (2.4)*
Clinical frailty scale, mean (SD)^a^	3.6 (1.3)
Non-frail (score 1–3)	43 (52.4)
Vulnerable or mildly frail (score 4–5)	32 (39.0)
Moderately or severely frail (score 6–7)	7 (8.5)
Charlson comorbidity index, median (IQR)^a^	4.0 (3.0–5.0)
CCI score 2-3^b^	31 (37.8)
CCI score ≥ 4	48 (58.5)
*Missing*	*3 (3.7)*
eGFR, *if not on dialysis,* mean (SD)	15.7 (4.3)
Started kidney replacement therapy	
Haemodialysis	2 (2.4)^c^
Peritoneal dialysis	0 (0.0)

The following continuous variables had missing data: eGFR at baseline (2.4%)

^a^These demographic and clinical characteristics were not reassessed during COVID-19 pandemic

^b^All patients scored minimum 2-points for *moderate to severe kidney disease*

^c^Two patients were already on haemodialysis treatment when having the follow-up measurement of POLDER study, which has been used as reference for outcomes before COVID-19 pandemic for the current study

### Concerns about COVID-19

**Fig. 2:**
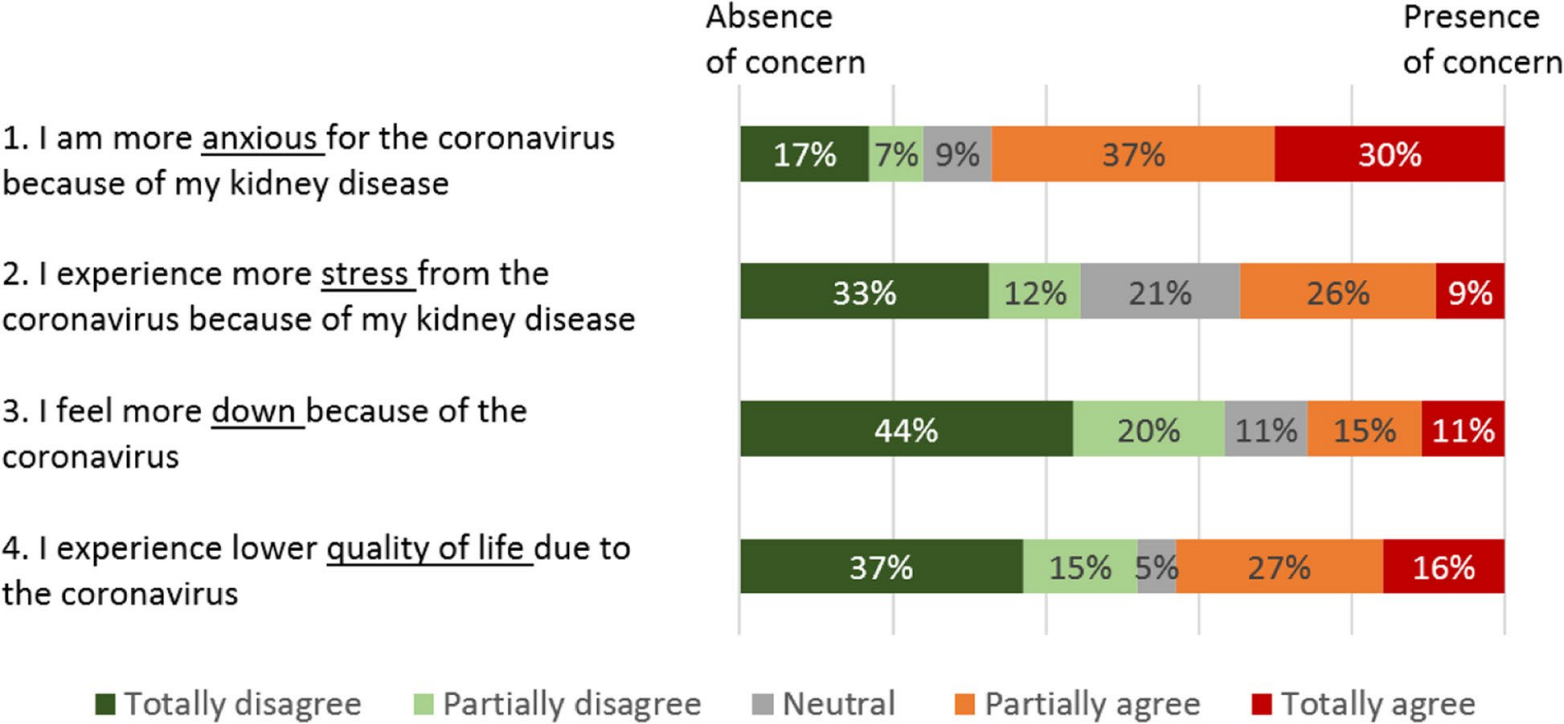
Respondents’ agreement to the COVID-19 related statement. Questions were scored on a scale from 1 ‘totally disagree’ to 5 ‘totally agree’

Figure 2 presents responses to the four COVID-19 related statements. The majority of the respondents (*n* = 55, 67%) reported to be more anxious about COVID-19 because of their kidney disease, while 43% (*n* = 35) stated that their quality of life was reduced because of the coronavirus. Furthermore, only 35% (*n* = 28) reported to experience more stress from the coronavirus because of their kidney disease, and 26% (*n* = 21) stated to feel more down due to the pandemic. Higher COVID-19-stress was associated with a lower education level (*p* = .036), and patients who reported to feel more down due to COVID-19 were more often female (*p* = .020). No other patient characteristics were associated with disease-related COVID-19-concerns, as shown in Additional file 1.

### General anxiety symptoms

The median HADS-A anxiety score was 3.0 (IQR 0.0–6.0, *n* = 80), as is shown in Table 2. In total, 15% of respondents (*n* = 12) exceeded the threshold (score ≥ 8) indicative for anxiety. Anxiety scores were higher among females compared to males (median 4.0 [IQR 3.0–9.0] versus 2.0 [0.0–6.0], *p* = .020), and weakly associated to a decline in eGFR (correlation coefficient .197, *p* = .023). No associations were found with other patient characteristics (Additional file 1).

**Table 2:** Mental wellbeing prior and during COVID-19 pandemic

	Score range^a^	n	Prior COVID-19 pandemic	During COVID-19 pandemic	*p*-value
***Cross-sectional outcomes***					
*Generic anxiety symptoms*					
HADS-A score, median (IQR)	0–21	80	–	3.0 (0.0–6.0)	
HADS-A score ≥ 8, n (%)		80	–	12 (15%)	
***Longitudinal outcomes***					
*Depressive symptoms*					
GDS-15 score, median (IQR)^b^	0–15	81	0.0 (0.0–3.0)	0.0 (0.0–4.0)	**.028**
GDS-15 score ≥ 5, n (%)	Yes/no	81	9 (11%)	18 (22%)	**.022**
*HRQoL*					
MCS, mean (SD)	0–100	80	50.3 (9.6)	50.4 (9.9)	.913
PCS, mean (SD)	0–100	80	40.4 (10.1)	36.1 (10.4)	**<.001**

*Abbreviations: GDS*-15 15-item geriatric depression scale, *HADS-A* anxiety subscale of hospital anxiety and depression scale, *HRQoL* health-related quality of life, *IQR* inter-quartile range, *MCS* mental component summary, *PCS* physical component summary, *SD* standard deviation

^a^Higher scores indicate more anxiety- or depressive symptoms, or better HRQoL

For *n* = 23 patients who scored positive on Whooley questions both before and during the pandemic

^b^Wher﻿e both Whooley questions were answered negative, we assumed GDS-15 score was 0

### Change in depressive symptoms

Table 2 shows that the presence of depressive symptoms (GDS-15 score ≥ 5) increased from 11% before the COVID-19 pandemic to 22% during the pandemic (*p* = .022). No patient characteristics were associated with changes in depressive symptoms (Additional file 2).

### Change in HRQoL

MCS did not change from before and during the COVID-19 pandemic (*M* = 50.3, *SD* = 9.6 to *M* = 50.4, *SD* = 9.9; *p* = .913; Table 2), and no associations were found with patient characteristics (Additional file 2). PCS decreased by − 4.3 (*M* = 40.4, *SD* = 10.1 to *M* = 36.1, *SD* = 10.4; *p* < .001). PCS changes were not associated with to patient characteristics except for sex (males showed a greater decline; − 5.3 (*SD* = 8.5) compared to − 0.9 (*SD* = 5.7) for females, *p* = .039).

### Change in emotional symptoms

**Fig. 3:**
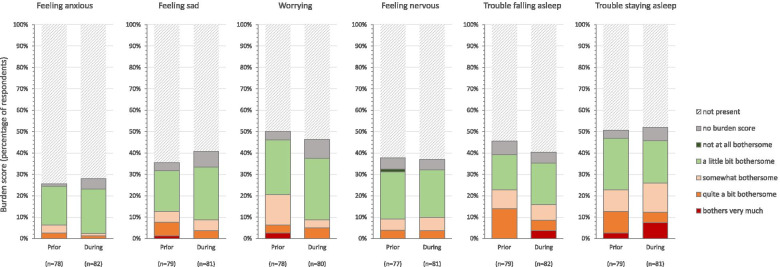
Symptom prevalence and burden score of six emotional symptoms (derived from DSI), prior to and during the COVID-19 pandemic. Patients indicated whether the symptom was present (yes/no) and, for present symptoms, they subsequently rated the burden on a 5-point Likert scale ranging from ‘not at all bothersome’ to ‘bothers very much’. Median (inter-quartile range) burden score for feeling anxious was 2.0 (2.0–3.0) prior to the pandemic and 2.0 (2.0–2.0, *p* = .227) during the pandemic, feeling sad 2.0 (2.0–3.5) and 2.0 (2.0–3.0, *p* = .314), worrying 2.0 (2.0–3.0) and 2.0 (2.0–2.3, *p* = .058), feeling nervous 2.0 (2.0–3.0) and 2.0 (2.0–3.0, *p* = .963), having trouble falling asleep 3.0 (2.0–4.0) and 2.0 (2.0–3.5, *p* = .594), having trouble staying asleep 2.0 (2.0–4.0) and 3.0 (2.0–4.0, *p* = .769)

During the COVID-19 pandemic, symptoms such as feeling anxious (*n* = 23, 28%), feeling sad (*n* = 33, 41%), and worrying (*n* = 37, 46%) were often reported by patients. When compared to before the COVID-19 pandemic, presence of these symptoms did not change (from 26 to 28%, *p* = .607; 35 to 41%, *p* = .227; 50 to 46%, *p* = 1.000; respectively). Other symptoms, which were present in one third to half of the population during the pandemic, did not change either compared to before COVID-19 pandemic (i.e. feeling nervous [prior 38% and during the pandemic 37%, *p* = 1.000], difficulty falling asleep [46 and 40%, *p* = .678] and staying asleep [51 and 52%, *p* = 1.000]). For all symptoms, the experienced burden did not change either (Fig. 3).

## Discussion

This study investigated mental wellbeing in older patients with advanced CKD (stage G4/G5) during the COVID-19 pandemic. The majority of patients reported to be more anxious about COVID-19 because of their kidney disease, and nearly half of them stated that their quality of life was reduced because of the pandemic. We found an increase in the presence of depressive symptoms and a modest decline in physical HRQoL during the COVID-19 pandemic compared to before. Mental HRQoL and emotional symptoms did not significantly change over time. Apart from increased general anxiety symptoms in females, and a faster decline in physical HRQoL in males, no other patient characteristics were associated with validated measures of mental wellbeing.

Our findings confirm the high prevalence of disease-related worries about COVID-19 among patients with other chronic conditions, varying from 40 to 56% in type 2 diabetes [31, 32] to 80% in cancer patients [33]. Interestingly, in our study, kidney disease-related COVID-19-anxiety was not associated with general anxiety symptoms (correlation coefficient .131, *p* = .139), suggesting that kidney disease-related COVID-19 anxiety does not directly translate into general anxiety symptoms such as restlessness, panic or tension. Also, we did not find higher HADS-A scores compared to previous studies in CKD G4/G5 patients [34, 35] and dialysis patients [21, 35]. Higher anxiety scores among females, one of our findings, has also been reported previously [35]. Having a male-predominant study population (77%), the anxiety scores of the study population as a whole might be an underestimation compared to populations with a more balanced sex ratio.

Regarding depressive feelings, 26% of our sample reported to feel more down because of the COVID-19 pandemic. Similarly, compared to pre-COVID-19, the presence of depressive symptoms increased significantly during the COVID-19 pandemic. However, prevalence of depression was lower compared to the average prevalence of clinical depression in CKD patients [10]. Since depression rates have been higher in dialysis patients compared to CKD population [10, 36], the start of dialysis treatment during the follow-up potentially influenced the increase in depressive symptoms.

Mental and physical HRQoL during the COVID-19 pandemic in our study were comparable with previous studies in CKD G4/G5 [11, 37] and the dialysis population [38, 39] prior to the pandemic. In line with results of these studies, physical HRQoL was lower than the general US and the older (aged 70–79 years) Dutch population [27, 40]. We found a modest decline in physical HRQoL (− 4.3), which could be considered borderline clinically relevant, since the minimal clinical important differences for physical HRQoL in CKD patients lies between 4.5 and 6.3 [41, 42]. Even though physical HRQoL was unrelated to eGFR or duration of follow-up, it is possible that disease progression influenced decline in physical HRQoL, as Wyld et al. [37] showed that patients with more advanced CKD experienced greater declines in physical HRQoL. We found a higher decline of physical HRQoL in males compared to females. At both time points scores were higher for males, particularly prior to the pandemic, which was consistent with other studies [37, 42]. As our study population consisted of more men than women, the decline in physical HRQoL for sex-balanced populations might be smaller.

Taken together, our results on mental wellbeing during COVID-19 are in line with previous pre-COVID-19 studies in CKD patients. In the general population, studies on mental wellbeing during COVID-19 show a similar picture, even though contradictory results have been found. On the one hand, absolute levels of anxiety, depression, mental health, and quality of life, during COVID-19 have been only temporarily affected or not affected at all [43, 44], whereas other studies have shown an increase [5] and persistence [6] in mental distress after the COVID-19 lockdown.

There are several potential explanations for our finding that the COVID-19 pandemic did not change the mental HRQoL or emotional symptoms of older CKD patients. First, government-imposed restrictions often restrict social gatherings and older patients are in general less socially active than younger patients. Additionally, economic uncertainty increases the risk of involuntary loss of income and older patients are often not or no longer employed. Several studies in the general population found more mental distress during the COVID-19 pandemic in both younger and employed people [5, 6, 45]. Second, the presence of a chronic disease may already have already had a larger effect on HRQoL of a patient than the added effect of COVID-19, as was suggested by Jeppensen et al. [33] Also, response shift may play a role, referring to a change over time in patient reported outcomes due to changes in internal standards of health and wellbeing, values and or reconceptualization of concepts such as HRQoL [46]. These cognitive changes reflect an adaptation to people’s situation and could explain why HRQoL during the pandemic may remain stable while physical health declines. Third, follow-up during COVID-19 pandemic may have been too short to detect HRQoL differences. Fourth, due to the broad concept of HRQoL, potential changes in mental wellbeing during COVID-19 may have been less prominent in HRQoL compared to anxiety and depressive symptoms.

Little is known about the implications of anxiety and depression during COVID-19 in the older CKD population. Further investigation of potential mental and physical consequences is therefore required, for example on physical activity and healthcare avoidance. Additionally, ongoing studies on mental health should take the effect of the COVID-19 pandemic into account. Studies have shown that restrictions on physical activity during COVID-19 pandemic have most frequently affected people aged ≥65 years [47], inactive individuals [48], and those with a low HRQoL before COVID-19 [49]. Furthermore, healthcare avoidance during the COVID-19 pandemic has been reported in cancer patients, among whom reluctance to consult medical care due to fear of COVID-19 infection varied between 9% [33] and 19% [50]. As worrying and less social support were shown to negatively impact HRQoL [14], and both anxiety and depression have been associated with adverse outcomes [36], there is an urgency for awareness of mental health problems and supporting patients’ needs during COVID-19 pandemic.

To our knowledge, this is the first study assessing mental wellbeing in older patients with CKD G4/G5 during the COVID-19 pandemic. Strengths of our study include the high response rate and the availability of multicentre baseline data before the COVID-19 outbreak. Our results should be interpreted in light of the limitations. First, our relatively small sample size, which most notably increases the risk of a type II error for the found absence of effect of the COVID-19 pandemic on both mental HRQoL and emotional symptoms. Second, the design of our study prevents us from drawing any causal conclusions about the effects of COVID-19 pandemic on mental wellbeing. Nonetheless, the prospective design allowed to assess changes in depression and physical HRQoL over time. Unfortunately, and this is our third limitation, for anxiety symptoms no comparison prior to the COVID-19 pandemic could be made. Fourth, ideally, data would have been collected during or shortly after the peak of the COVID-19 infections in the Netherlands. Yet, the questionnaire was sent early June, when infection rates were stable enough to lift the stringent lockdown measures. However, most questions refer to the prior month, hereby also including the lockdown period. Still, recall bias may have affected these results.

## Conclusions

In conclusion, our findings show that older patients with advanced CKD suffered from disease-related anxiety about COVID-19, increased depressive symptoms, and reduced physical HRQoL during the COVID-19 pandemic. No effect on mental HRQoL or emotional symptoms was found. The impact of the pandemic on this vulnerable patient group extends beyond increased mortality risk, and awareness of mental health problems during the pandemic is essential. More in-depth investigation on disease-related COVID-19 concerns and its implications for the CKD population is needed.

## Supplementary Information

**Additional file 1 Table 1**. Correlations of baseline characteristics and cross-sectional outcomes of mental wellbeing.

**Additional file 2 Table 2**. Correlations of baseline characteristics and change in longitudinal outcomes of mental wellbeing.

**Additional file 3.** Group information for the Pathway for older patients reaching end stage renal disease (POLDER) study group.

## Data Availability

The datasets generated and/or analysed during the current study are not publicly available due to the privacy of individuals that participated in the study, but are available from the corresponding author on reasonable request.

## Abbreviations

CKD: Chronic kidney disease
CKD stage G4/G5: Chronic kidney disease with severe decreased glomerular filtration rate or kidney failure
COVID-19: Coronavirus 2019
DSI: Dialysis Symptom Index
eGFR: Estimated glomerular filtration rate
GDS-15: Geriatric Depression Scale 15-item
HADS-A: Hospital Anxiety and Depression Scale-anxiety subscale
HRQoL: Health-related quality of life
IQR: Inter-quartile range
M: Mean
MCS: Mental Component Summary (measure of HRQoL)
NTR: Netherlands Trial Register
PCS: Physical Component Summary (measure of HRQoL)
POLDER: Pathway for OLder patients reaching End-stage Renal disease
SD: Standard deviation
SF-12: Short Form Health Survey 12-item (measure of HRQoL)

## References

[1] Williamson EJ, Walker AJ, Bhaskaran K, Bacon S, Bates C, Morton CE (2020). Factors associated with COVID-19-related death using OpenSAFELY. Nature.

[2] Luo M, Guo L, Yu M, Jiang W, Wang H (2020). The psychological and mental impact of coronavirus disease 2019 (COVID-19) on medical staff and general public - a systematic review and meta-analysis. Psychiatry Res.

[3] Pappa S, Ntella V, Giannakas T, Giannakoulis VG, Papoutsi E, Katsaounou P (2020). Prevalence of depression, anxiety, and insomnia among healthcare workers during the COVID-19 pandemic: a systematic review and meta-analysis. Brain Behav Immun.

[4] Wang C, Pan R, Wan X, Tan Y, Xu L, Ho CS (2020). Immediate psychological responses and associated factors during the initial stage of the 2019 coronavirus disease (COVID-19) epidemic among the general population in China. Int J Environ Res Public Health.

[5] Pierce M, Hope H, Ford T, Hatch S, Hotopf M, John A (2020). Mental health before and during the COVID-19 pandemic: a longitudinal probability sample survey of the UK population. Lancet Psychiatry.

[6] McGinty EE, Presskreischer R, Anderson KE, Han H, Barry CL (2020). Psychological distress and COVID-19-related stressors reported in a longitudinal cohort of US adults in April and July 2020. JAMA.

[7] Berg-Weger M, Morley JE (2020). Editorial: loneliness and social isolation in older adults during the COVID-19 pandemic: implications for Gerontological social work. J Nutr Health Aging.

[8] Brooke J, Jackson D (2020). Older people and COVID-19: isolation, risk and ageism. J Clin Nurs.

[9] Santini ZI, Jose PE, York Cornwell E, Koyanagi A, Nielsen L, Hinrichsen C (2020). Social disconnectedness, perceived isolation, and symptoms of depression and anxiety among older Americans (NSHAP): a longitudinal mediation analysis. Lancet Public Health.

[10] Palmer S, Vecchio M, Craig JC, Tonelli M, Johnson DW, Nicolucci A (2013). Prevalence of depression in chronic kidney disease: systematic review and meta-analysis of observational studies. Kidney Int.

[11] Voskamp PWM, van Diepen M, Evans M, Caskey FJ, Torino C, Postorino M (2019). The impact of symptoms on health-related quality of life in elderly pre-dialysis patients: effect and importance in the EQUAL study. Nephrol Dial Transplant.

[12] Mujais SK, Story K, Brouillette J, Takano T, Soroka S, Franek C (2009). Health-related quality of life in CKD patients: correlates and evolution over time. Clin J Am Soc Nephrol.

[13] Lee YJ, Kim MS, Cho S, Kim SR (2013). Association of depression and anxiety with reduced quality of life in patients with predialysis chronic kidney disease. Int J Clin Pract.

[14] Tommel J, Evers AWM, van Hamersvelt HW, Jordens R, van Dijk S, Hilbrands LB, et al. Predicting health-related quality of life in dialysis patients: factors related to negative outcome expectancies and social support. Patient Educ Couns. 2020. 10.1016/j.pec.2020.11.019. 10.1016/j.pec.2020.11.019 33293180

[15] Shirazian S, Grant CD, Aina O, Mattana J, Khorassani F, Ricardo AC (2017). Depression in chronic kidney disease and end-stage renal disease: similarities and differences in diagnosis, epidemiology, and management. Kidney Int Rep.

[16] Farrokhi F, Abedi N, Beyene J, Kurdyak P, Jassal SV (2014). Association between depression and mortality in patients receiving long-term dialysis: a systematic review and meta-analysis. Am J Kidney Dis.

[17] Charlson ME, Pompei P, Ales KL, MacKenzie CR (1987). A new method of classifying prognostic comorbidity in longitudinal studies: development and validation. J Chronic Dis.

[18] Rockwood K, Song X, MacKnight C, Bergman H, Hogan DB, McDowell I (2005). A global clinical measure of fitness and frailty in elderly people. Cmaj.

[19] Zigmond AS, Snaith RP (1983). The hospital anxiety and depression scale. Acta Psychiatr Scand.

[20] Spinhoven P, Ormel J, Sloekers PP, Kempen GI, Speckens AE, Van Hemert AM (1997). A validation study of the hospital anxiety and depression scale (HADS) in different groups of Dutch subjects. Psychol Med.

[21] Loosman WL, Siegert CE, Korzec A, Honig A (2010). Validity of the hospital anxiety and depression scale and the Beck depression inventory for use in end-stage renal disease patients. Br J Clin Psychol.

[22] Olssøn I, Mykletun A, Dahl AA (2005). The hospital anxiety and depression rating scale: a cross-sectional study of psychometrics and case finding abilities in general practice. BMC Psychiatry.

[23] Whooley MA, Avins AL, Miranda J, Browner WS (1997). Case-finding instruments for depression. Two questions are as good as many. J Gen Intern Med.

[24] Sheikh JI, Yesavage JA (1986). Geriatric depression scale (GDS): recent evidence and development of a shorter version. Clin Gerontol.

[25] Tsoi KK, Chan JY, Hirai HW, Wong SY (2017). Comparison of diagnostic performance of two-question screen and 15 depression screening instruments for older adults: systematic review and meta-analysis. Br J Psychiatry.

[26] Dennis M, Kadri A, Coffey J (2012). Depression in older people in the general hospital: a systematic review of screening instruments. Age Ageing.

[27] Ware J, Kosinski M, Keller SD (1996). A 12-item short-form health survey: construction of scales and preliminary tests of reliability and validity. Med Care.

[28] Loosman WL, Hoekstra T, van Dijk S, Terwee CB, Honig A, Siegert CE (2015). Short-form 12 or short-form 36 to measure quality-of-life changes in dialysis patients?. Nephrol Dial Transplant.

[29] Weisbord SD, Fried LF, Arnold RM, Rotondi AJ, Fine MJ, Levenson DJ (2004). Development of a symptom assessment instrument for chronic hemodialysis patients: the Dialysis symptom index. J Pain Symptom Manag.

[30] Eekhout I, de Vet HC, Twisk JW, Brand JP, de Boer MR, Heymans MW (2014). Missing data in a multi-item instrument were best handled by multiple imputation at the item score level. J Clin Epidemiol.

[31] Joensen LE, Madsen KP, Holm L, Nielsen KA, Rod MH, Petersen AA (2020). Diabetes and COVID-19: psychosocial consequences of the COVID-19 pandemic in people with diabetes in Denmark-what characterizes people with high levels of COVID-19-related worries?. Diabet Med.

[32] Nachimuthu S, Vijayalakshmi R, Sudha M, Viswanathan V (2020). Coping with diabetes during the COVID - 19 lockdown in India: results of an online pilot survey. Diabetes Metab Syndr.

[33] Jeppesen SS, Bentsen KK, Jørgensen TL, Holm HS, Holst-Christensen L, Tarpgaard LS, et al. Quality of life in patients with cancer during the COVID-19 pandemic - a Danish cross-sectional study (COPICADS). Acta Oncol. 2020. 10.1080/0284186x.2020.1830169. 10.1080/0284186X.2020.1830169 33031010

[34] Bezerra CIL, Silva BC, Elias RM (2018). Decision-making process in the pre-dialysis CKD patients: do anxiety, stress and depression matter?. BMC Nephrol.

[35] Shafi ST, Shafi T (2017). A comparison of anxiety and depression between pre-dialysis chronic kidney disease patients and hemodialysis patients using hospital anxiety and depression scale. Pak J Med Sci.

[36] Schouten RW, Haverkamp GL, Loosman WL, Chandie Shaw PK, van Ittersum FJ, Smets YFC (2019). Anxiety symptoms, mortality, and hospitalization in patients receiving maintenance Dialysis: a cohort study. Am J Kidney Dis.

[37] Wyld MLR, Morton RL, Clayton P, Wong MG, Jardine M, Polkinghorne K (2019). The impact of progressive chronic kidney disease on health-related quality-of-life: a 12-year community cohort study. Qual Life Res.

[38] Ware JE, Richardson MM, Meyer KB, Gandek B (2019). Improving CKD-specific patient-reported measures of health-related quality of life. J Am Soc Nephrol.

[39] van der Willik EM, Hemmelder MH, Bart HA, van Ittersum FJ, Hoogendijk-van den Akker JM, WJW B, et al. Routinely measuring symptom burden and health-related quality of life in dialysis patients: first results from the Dutch registry of patient-reported outcome measures. Clin Kidney J. 2021;14:1535–44. 10.1093/ckj/sfz192 PMC8286800 34285801

[40] Mols F, Pelle AJ, Kupper N (2009). Normative data of the SF-12 health survey with validation using postmyocardial infarction patients in the Dutch population. Qual Life Res.

[41] Erez G, Selman L, Murtagh FE (2016). Measuring health-related quality of life in patients with conservatively managed stage 5 chronic kidney disease: limitations of the medical outcomes study short form 36: SF-36. Qual Life Res.

[42] Legrand K, Speyer E, Stengel B, Frimat L, Ngueyon Sime W, Massy ZA (2020). Perceived health and quality of life in patients with CKD, including those with kidney failure: findings from National Surveys in France. Am J Kidney Dis.

[43] Clotworthy A, Dissing AS, Nguyen TL, Jensen AK, Bilsteen JF, Andersen TO (2020). 'Standing together - at a distance': documenting changes in mental-health indicators in Denmark during the COVID-19 pandemic. Scand J Public Health.

[44] van der Velden PG, Contino C, Das M, van Loon P, Bosmans MWG (2020). Anxiety and depression symptoms, and lack of emotional support among the general population before and during the COVID-19 pandemic. A prospective national study on prevalence and risk factors. J Affect Disord.

[45] Pieh C, Budimir S, Probst T (2020). The effect of age, gender, income, work, and physical activity on mental health during coronavirus disease (COVID-19) lockdown in Austria. J Psychosom Res.

[46] van der Willik EM, Terwee CB, Bos WJW, Hemmelder MH, Jager KJ, Zoccali C, et al. Patient-reported outcome measures (PROMs): making sense of individual PROM scores and changes in PROM scores over time. Nephrology (Carlton). 2020. 10.1111/nep.13843. 10.1111/nep.13843 PMC8048666 33325638

[47] Heid AR, Cartwright F, Wilson-Genderson M, Pruchno R. Challenges experienced by older people during the initial months of the COVID-19 pandemic. Gerontologist. 2020. 10.1093/geront/gnaa138. 10.1093/geront/gnaa138 PMC7543473 32955079

[48] Lesser IA, Nienhuis CP (2020). The impact of COVID-19 on physical activity behavior and well-being of Canadians. Int J Environ Res Public Health.

[49] Suzuki Y, Maeda N, Hirado D, Shirakawa T, Urabe Y (2020). Physical activity changes and its risk factors among community-dwelling Japanese older adults during the COVID-19 epidemic: associations with subjective well-being and health-related quality of life. Int J Environ Res Public Health.

[50] de Joode K, Dumoulin DW, Engelen V, Bloemendal HJ, Verheij M, van Laarhoven HWM (2020). Impact of the coronavirus disease 2019 pandemic on cancer treatment: the patients' perspective. Eur J Cancer.

