# Supplementary material for: Impact of the COVID-19 pandemic on symptoms of anxiety and depression and health-related quality of life in older patients with chronic kidney disease
Source: BMC Geriatr. 2021 Nov 19;21:650. doi: 10.1186/s12877-021-02593-0 (PMC8602979; doi:10.1186/s12877-021-02593-0)
Supplement: Supplementary file 1 — Additional file 1 Table 1. Correlations of baseline characteristics and cross-sectional outcomes of mental wellbeing. [file 12877_2021_2593_MOESM1_ESM.docx]

**Impact of the COVID-19 pandemic on symptoms of anxiety and depression and health-related quality of life in older patients with chronic kidney disease.**

C.G.N.Voorend*, M. van Oevelen, M. Nieberg, Y. Meuleman, C.F.M. Franssen, H. Joosten, N. Berkhout, A.C. Abrahams, S.P. Mooijaart, W.J.W. Bos, M. van Buren, on behalf of the POLDER investigators

* corresponding author: Leiden University Medical Center, [c.g.n.voorend@lumc.nl](mailto:c.g.n.voorend@lumc.nl)

**Additional table 1. Correlations of baseline characteristics and cross-sectional outcomes of mental wellbeing.**

|  | **Kidney disease-related concerns for COVID-19**** | | | | | | | | | | |  | **Anxiety symptoms** | |  |
| --- | --- | --- | --- | --- | --- | --- | --- | --- | --- | --- | --- | --- | --- | --- | --- |
|  | ***anxious***  (n = 82) | |  | ***stress***  (n = 82) | |  | ***feeling down***  (n = 82) | |  | ***quality of life***  (n = 81) | |  | HADS-A  (n = 80) | |  |
|  | Correlation coefficient | *p*-value |  | Correlation coefficient | *p*-value |  | Correlation coefficient | *p*-value |  | Correlation coefficient | *p*-value |  | Correlation  coefficient | *p*-value | |
| Age | -.135 | .106 |  | -.029 | .725 |  | -.032 | .701 |  | -.020 | .808 |  | .077 | .335 | |
| Sex | NA | .558 |  | NA | .074 |  | **NA** | **.020** |  | NA | .546 |  | **NA** | **.020** | |
| Living status* | NA | .734 |  | NA | .224 |  | NA | .147 |  | NA | .431 |  | NA | .091 | |
| Level of education* | NA | .162 |  | **NA** | **.036** |  | NA | .102 |  | NA | .889 |  | NA | .219 | |
| Clinical frailty scale* | .058 | .527 |  | .081 | .369 |  | .126 | .166 |  | .013 | .885 |  | .064 | .506 | |
| Charlson comorbidity index * | .171 | .063 |  | .100 | .270 |  | .041 | .657 |  | -.073 | .428 |  | -.114 | .194 | |
| eGFR, *if not on dialysis* | -.096 | .295 |  | -.094 | .298 |  | .072 | .431 |  | -.010 | .913 |  | .097 | .264 | |
| Kidney replacement therapy | NA | .750 |  | NA | .288 |  | NA | .465 |  | NA | .091 |  | NA | .584 | |
| Follow-up duration | -.107 | .200 |  | -.031 | .706 |  | -.111 | .183 |  | -.004 | .966 |  | .004 | .963 | |

* Measured at baseline,

** Four statements (i.e. ‘I am more anxious for the coronavirus because of my kidney disease’, ‘I experience more stress from the coronavirus because of my kidney disease’, ‘I feel more down because of the coronavirus’, and ‘I experience a lower quality of life due to the coronavirus’) for which agreement was rated on a five-point Likert scale.

The following baseline variables had missing data: Living status (1.2%), Level of education (2.4%), Charlson comorbidity index (3.7%).
Abbreviations: HADS-A, Anxiety component of the Hospital Anxiety and Depression scale; NA, not applicable
