# Supplementary material for: Impact of the COVID-19 pandemic on symptoms of anxiety and depression and health-related quality of life in older patients with chronic kidney disease
Source: BMC Geriatr. 2021 Nov 19;21:650. doi: 10.1186/s12877-021-02593-0 (PMC8602979; doi:10.1186/s12877-021-02593-0)
Supplement: Supplementary file 2 — Additional file 2 Table 2. Correlations of baseline characteristics and change in longitudinal outcomes of mental wellbeing. [file 12877_2021_2593_MOESM2_ESM.docx]

**Impact of the COVID-19 pandemic on symptoms of anxiety and depression and health-related quality of life in older patients with chronic kidney disease.**

C.G.N.Voorend*, M. van Oevelen, M. Nieberg, Y. Meuleman, C.F.M. Franssen, H. Joosten, N. Berkhout, A.C. Abrahams, S.P. Mooijaart, W.J.W. Bos, M. van Buren, on behalf of the POLDER investigators

* corresponding author: Leiden University Medical Center, [c.g.n.voorend@lumc.nl](mailto:c.g.n.voorend@lumc.nl)

**Additional Table 2. Correlations of baseline characteristics and change in longitudinal outcomes of mental wellbeing.**

|  | **Mental HRQoL**  ∆ MCS  (n = 80) | |  | **Physical HRQoL**  ∆ PCS  (n =80) | |  | **Depressive symptoms**  ∆ GDS-15 **  (n = 81) | | |
| --- | --- | --- | --- | --- | --- | --- | --- | --- | --- |
|  | **Correlation**  **coefficient** | ***p*-value** |  | **Correlation**  **coefficient** | ***p*-value** |  | **Correlation**  **coefficient** | | ***p*-value** |
| Age | -.049 | .670 |  | -.035 | .759 |  | -.043 | .606 | |
| Sex | NA | .126 |  | **NA** | **.039** |  | NA | .336 | |
| Living status* | NA | .541 |  | NA | .449 |  | NA | .842 | |
| Level of education | NA | .841 |  | NA | .577 |  | NA | .275 | |
| Clinical frailty scale* | .029 | .800 |  | .188 | .098 |  | .138 | .170 | |
| Charlson comorbidity index * | .233 | .042 |  | -.043 | .716 |  | -.134 | .141 | |
| eGFR, *if not on dialysis* | -.049 | .694 |  | .117 | .342 |  | -.026 | .774 | |
| Kidney replacement therapy | NA | .234 |  | NA | .559 |  | NA | .943 | |
| Follow-up duration | -.073 | .535 |  | -.005 | .966 |  | .039 | .637 | |

* Measured at baseline, ** Patients who scored zero positive Whooley questions had a GDS-15 score of ‘0’.

Abbreviations: GDS-15, Geriatric Depression Scale 15-item; HRQoL, health-related quality of life; MCS, mental component summary; NA, not applicable; PCS, physical component summary.
