# Supplementary material for: Impact of the COVID-19 pandemic on symptoms of anxiety and depression and health-related quality of life in older patients with chronic kidney disease
Source: BMC Geriatr. 2021 Nov 19;21:650. doi: 10.1186/s12877-021-02593-0 (PMC8602979; doi:10.1186/s12877-021-02593-0)
Supplement: Supplementary file 3 — Additional file 3. Group information for the Pathway for older patients reaching end stage renal disease (POLDER) study group. [file 12877_2021_2593_MOESM3_ESM.docx]

**Impact of the COVID-19 pandemic on symptoms of anxiety and depression and health-related quality of life in older patients with chronic kidney disease.**

C.G.N.Voorend*, M. van Oevelen, M. Nieberg, Y. Meuleman, C.F.M. Franssen, H. Joosten, N. Berkhout, A.C. Abrahams, S.P. Mooijaart, W.J.W. Bos, M. van Buren, on behalf of the POLDER investigators

* corresponding author: Leiden University Medical Center, [c.g.n.voorend@lumc.nl](mailto:c.g.n.voorend@lumc.nl)

**Additional file 3. Group information for the Pathway for older patients reaching end stage renal disease (POLDER) study group**

The Pathway for older patients reaching end stage renal disease (POLDER) study group is a collaboration in the Netherlands that is established to study and implement a nephrology-tailored geriatric assessment in routine care.

The POLDER investigators are (in alphabetical order): Arjan van Alphen, Maasstad Hospital Rotterdam; Noeleen Berkhout-Byrne, Leiden University Medical Center; G.F (Fenna) van Breda, Amsterdam University Medical Center; Marjolijn van Buren, Haga Teaching Hospital The Hague; Henk Boom, Reiner de Graaf Hospital Delft; Willem Jan Bos, St. Antonius Hospital Nieuwegein; Adry Diepenbroek, University Medical Center Groningen; Marielle Emmelot-Vonk, University Medical Center Utrecht; Casper Franssen, University Medical Center Groningen; Carlo AJM Gaillard, University Medical Center Utrecht; Nel Groeneweg-Peeters, Reinier de Graaf Hospital Delft; Bettie Hoekstra, Maasstad Hospital Rotterdam; Nienke Hommes, Haaglanden Medical Center The Hague; Francoise Hoornaar, St. Antonius Hospital Nieuwegein; Hanneke Joosten, Maastricht University Medical Center; Joep Lagro, Haga Teaching Hospital; Elisabeth Litjens, Maastricht University Medical Center; Femke Molenaar, University Medical Center Utrecht; Simon P Mooijaart, Leiden University Medical Center; Aegida Neradova, Amsterdam University Medical Center, Dianet Amsterdam; Mike Peters, Amsterdam University Medical Center; Wilma Veldman, University Medical Center Groningen; Carlijn Voorend, Leiden University Medical Center; Lidwien Westerbos, Amsterdam University Medical Center; Carlijne Westerman- van der Wijden, Haaglanden Medical Center The Hague; Judith Wierdsma, University Medical Center Utrecht.
